# Detection of Autochthonous Zika Virus Transmission in Sincelejo, Colombia

**DOI:** 10.3201/eid2205.160023

**Published:** 2016-05

**Authors:** Erwin Camacho, Margaret Paternina-Gomez, Pedro J. Blanco, Jorge E. Osorio, Matthew T. Aliota

**Affiliations:** University of Wisconsin–Madison, Madison, Wisconsin, USA (E. Camacho, J.E. Osorio, M.T. Aliota);; Universidad de Sucre, Sincelejo, Colombia (M. Paternina-Gomez, P.J. Blanco)

**Keywords:** Sincelejo, Colombia, Zika virus, detection, diagnosis, outbreak, arbovirus, *Aedes aegypti*, vector-borne infections, mosquito, viruses

**To the Editor:** Zika virus is an arthropodborne member of the genus *Flavivirus* of the Spondweni serocomplex and is transmitted by *Aedes* mosquitoes (primarily *Ae. aegypti* in urban and periurban cycles). Zika virus emerged in Africa and has caused outbreaks of febrile disease that clinically resemble dengue fever and other arboviral diseases (*1*) but has been linked to neurologic syndromes and congenital malformation (*2*). Outbreaks have been reported in the Yap islands of the Federated States of Micronesia (*3*), French Polynesia (*4*), and Oceania; Brazil is currently experiencing the first reported local transmission of Zika virus in the Americas (*5*).

The future spread of Zika virus is unpredictable, but the history of the virus has been reminiscent of chikungunya virus (CHIKV), which reemerged in Africa and now circulates on all inhabited continents and is a major global health problem. Zika virus has been found in Colombia and is likely following the path of CHIKV, which reached the country in August 2014 (*6*). The virus co-circulates with other *Ae. aegypti*–transmitted arboviruses, including dengue virus (DENV) and yellow fever virus. We report Zika virus infection in Colombia and a recent ongoing outbreak in Sincelejo, Colombia, with resulting illness characterized by maculopapular rash, fever, myalgia/arthralgia, and conjunctivitis.

During October–November 2015, a total of 22 patients received a presumptive diagnosis of an acute viral illness by emergency department physicians at the Centro de Diagnostico Medico-Universidad de Sucre in Sincelejo. The patients began treatment for a dengue-like illness, and blood samples were obtained for diagnosis. The samples were analyzed at the Universidad de Sucre by using reverse transcription PCR (RT-PCR) to detect DENV, CHIKV, or Zika virus. Viral RNA was extracted from the serum samples by using the ZR Viral RNA Kit (Zymo Research, Irvine, CA, USA); reverse transcription was performed by using the Protoscript First Strand cDNA Synthesis Kit (New England Biolabs, Ipswich, MA, USA). Amplicons were produced by using the OneTaq Quick-Load 2X Master Mix (New England Biolabs) with primers specific to DENV (*7*), CHIKV (forward: 5′-CGCCAACATTCTGCTTACAC-3′; reverse: 5′-AGGATGCCGGTCATTTGAT-3′), and Zika virus. The CHIKV amplification target was 649 bp of nonstructural protein 1 (NS1). A positive PCR for a partial region of the envelope (E) gene with primers ZIKVENVF and ZIKVENVR (*8*) was considered indicative of Zika virus infection. Zika virus primers specific for the E gene and NS1 were designed and used to amplify the E gene and NS1 for phylogenetic analyses, and amplicons were produced by using the OneTaq One-Step RT-PCR Kit (New England Biolabs). E gene and NS1 PCR products were sequenced at the University of Wisconsin–Madison Biotechnology Center (Madison, WI, USA).

Samples from all patients were negative by RT-PCR for DENV and CHIKV; samples from 9 (41%) patients were positive for Zika virus. Among those 9 patients, 7 (78%) were male; median age was 23; and none had a history of international travel. Zika virus was analyzed by sequencing the E gene and NS1 of 2 isolates. Phylogenetic analyses rooted with Spondweni virus showed that the Zika virus sequences (GenBank accession nos. KU646827 and KU646828) belonged to the Asian lineage (Figure) and were closely related to strains isolated during the 2015 outbreak in Brazil (*5*). The sequences also showed 99% identity with sequences from a Zika virus isolate from French Polynesia (GenBank accession no. KJ776791) (*9*). These data suggest that Zika virus circulating in Colombia could have been imported from Brazil, most likely as a result of tourism activities on Colombia’s northern coast, where the first reported case was identified (the state of Bolivar).

**Figure:**
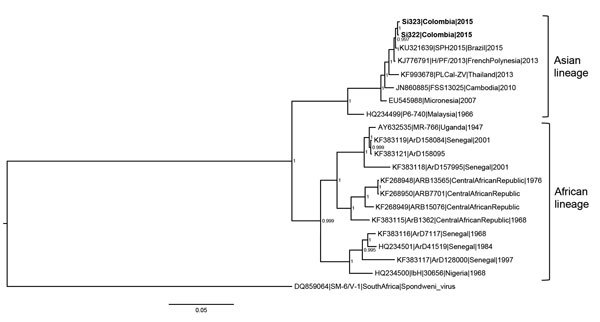
Majority-rule consensus tree based on Zika virus envelope and nonstructural protein 1 gene sequences (2,604 nt) of isolates from patients in Sincelejo, Colombia, October–November 2015, compared with reference isolates. The tree was constructed on the basis of Bayesian phylogenetic analysis with 8 million generations and a general time-reversible substitution model using MrBayes software version 3.2 (http://mrbayes.sourceforge.net). Numbers to the right of nodes represent posterior probabilities for corresponding clades. Samples sequenced in this study are in bold, and sequences are listed with GenBank accession numbers and are coded as accession no./strain/country/year of isolation when all information was available. The Colombia sequences are grouped with the Asian lineage of Zika virus. The tree was rooted with the Spondweni virus isolated in South Africa as the outgroup. Scale bar indicates nucleotide substitutions per site.

We report Zika virus infection in Colombia in association with an ongoing outbreak of acute maculoexantematic illness. Since detection of Zika virus in Sincelejo, a total of 13,500 cases have been identified in 28 of the country’s 32 territorial entities (*10*), all of which have abundant populations of *Ae. aegypti* mosquitoes and co-circulation of DENV and CHIKV. These circumstances highlight the need for accurate laboratory diagnostics and suggest that monitoring whether the virus spreads into neighboring countries (e.g., Ecuador, Peru, Venezuela, and Panama) is imperative.
